# A novel endoscopic electrocoagulation hemostasis technique for uncontrolled intraprocedural bleeding: series connection of foreign body forceps and hemostatic forceps

**DOI:** 10.1055/a-2291-9766

**Published:** 2024-04-09

**Authors:** Ruide Liu, Xianglei Yuan, Jia Liu, Shuang Liu, Jia Xie, Bing Hu

**Affiliations:** 1 34753 Department of Gastroenterology and Hepatology & Digestive Endoscopy Medical Engineering Research Laboratory West China Hospital, Sichuan University, Chengdu, Sichuan, China

Intraprocedural bleeding is unavoidable during endoscopic procedures 1 2 . Minor bleeding from small vessels can typically be managed with an electric knife, while significant bleeding from larger vessels requires the use of hemostatic forceps 3 4 . In cases of uncontrollable severe bleeding, clips can be used as rescue treatment, but their use may impede subsequent procedures. Here, we introduce a method using foreign body forceps and hemostatic forceps to achieve hemostasis for emergency massive bleeding during endoscopic full-thickness resection (EFTR).

A 72-year-old woman was referred to our hospital for the treatment of a gastric mass. Endoscopy, endoscopic ultrasound, and upper abdominal enhanced computed tomography were performed ( Fig. 1 ). Following consultation with the patient and her family, EFTR was chosen as the optimal treatment approach.

**Fig. 1:**
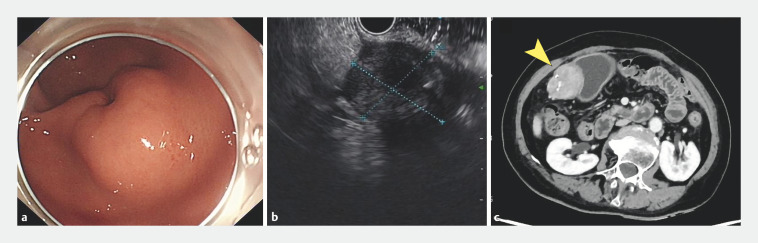
Examinations before endoscopic full-thickness resection of a gastric mass in a 72-year-old woman. **a** Endoscopy showed a raised lesion with a central depression at the gastric antrum–body junction. **b** Endoscopic ultrasound demonstrated a hypoechoic mass about 3.1 × 3.5 cm in size, with heterogeneous anechoic areas, originating from the muscularis propria layer. **c** Upper abdominal enhanced computed tomography showed a soft tissue density mass measuring 4.4 × 3.6 cm, with calcification in the gastric antrum, protruding into the abdominal cavity (yellow arrowhead).

During the procedure, sudden bleeding occurred that could not be controlled using the electric knife ( Video 1 , Fig. 2 **a** ). Despite repeated attempts with hemostatic forceps (FG-44NR-1; Olympus, Tokyo), bleeding persisted. Given the potential depth of the bleeding site and the longer jaws of foreign body forceps (4.5 mm, FD-410LR; Olympus) compared to hemostatic forceps (3.6 mm), we used a foreign body forceps to clamp the bleeding site, despite its lack of electrocoagulation capabilities. We then extracorporeally connected the foreign body forceps in series with a hemostatic forceps ( Fig. 2 **b** ). This enabled charging of the distal end of the foreign body forceps and successful hemostasis of the bleeding site ( Fig. 2 **c** , **d** ).

A novel endoscopic electrocoagulation technique for hemostasis of uncontrolled intraprocedural bleeding: series connection of foreign body forceps and hemostatic forceps.Video 1

**Fig. 2:**
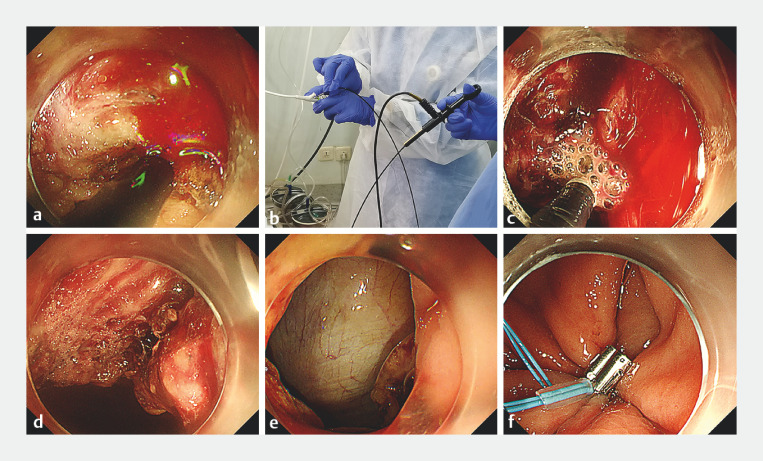
Series connection of foreign body forceps and hemostatic forceps for hemostasis of uncontrolled intraprocedural bleeding. **a** Sudden bleeding occurred during an endoscopic full-thickness resection, which could not be controlled using the electric knife and hemostatic forceps. **b** Extracorporeal connection of a hemostatic forceps in series with the foreign body forceps that was clamping the bleeding site. **c** This series connection enabled the distal end of the foreign body forceps to be charged, thereby providing electrocoagulation functionality. **d** This enabled successful hemostasis of the sudden bleeding. **e** The lesion was completely resected. **f** The wound was closed with a pursestring suture of clips and nylon ring.

Finally, the lesion was completely removed, and the wound was closed with a pursestring suture of clips and nylon ring ( Fig. 2 **e** , **f** ). Postoperative recovery was uneventful, with no rebleeding. Histopathology revealed a gastric schwannoma 5 ( Fig. 3 ).

**Fig. 3:**
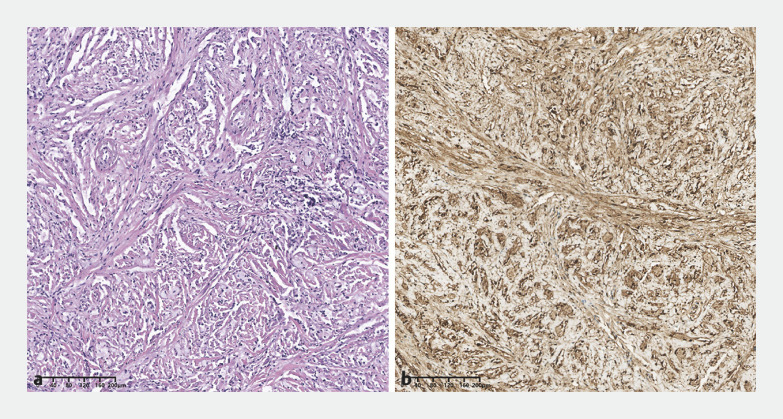
Histopathology of postoperative specimen. **a** Histology showed a spindle-cell tumor (hematoxylin and eosin, × 100). **b** Immunohistochemical staining of S-100 was positive, confirming the diagnosis of a gastric schwannoma (× 100).

This connection in series of forceps to enable hemostasis highlights the adaptability of endoscopic equipment and the imaginative innovation on the part of the endoscopist, providing a novel approach for managing uncontrollable intraprocedural bleeding without interrupting the operation.

Endoscopy_UCTN_Code_TTT_1AO_2AD

## Notes

Endoscopy E-Videos https://eref.thieme.de/e-videos
*E-Videos* is an open access online section of the journal *Endoscopy* , reporting on interesting cases and new techniques in gastroenterological endoscopy. All papers include a high-quality video and are published with a Creative Commons CC-BY license. Endoscopy E-Videos qualify for HINARI discounts and waivers and eligibility is automatically checked during the submission process. We grant 100% waivers to articles whose corresponding authors are based in Group A countries and 50% waivers to those who are based in Group B countries as classified by Research4Life (see: https://www.research4life.org/access/eligibility/ ). This section has its own submission website at https://mc.manuscriptcentral.com/e-videos .
